# The psychometric properties of the Migraine-Specific Quality of Life Questionnaire version 2.1 (MSQ) in chronic migraine patients

**DOI:** 10.1007/s11136-012-0230-7

**Published:** 2012-07-15

**Authors:** Regina Rendas-Baum, Lisa M. Bloudek, Gregory A. Maglinte, Sepideh F. Varon

**Affiliations:** 1 QualityMetric, Inc., 24 Albion Road, Bldg 400, Lincoln, RI 02865-4207 USA; 2 Allergan, Inc., Irvine, CA USA; 3 Amgen, Inc., Thousand Oaks, CA USA

**Keywords:** Migraine-Specific Quality of Life Questionnaire (MSQ), Psychometric properties, Validity, Chronic migraine, Health-related quality of life, PREEMPT

## Abstract

**Objective:**

The Migraine-Specific Quality of Life Questionnaire version 2.1 (MSQ) has been shown to have good psychometric performance in measuring headache impact in migraine patients, but its properties specifically in chronic migraine (CM) patients are unknown. The objective of this study was to evaluate the psychometric properties of the MSQ in a group of CM patients undergoing prophylactic treatment.

**Methods:**

Measurement properties of the MSQ were examined using two international, multicenter, randomized clinical trials evaluating onabotulinumtoxinA as headache prophylaxis in CM patients (*N* = 1,376). Confirmatory factor analysis (CFA) was used to test the latent structure of the MSQ in CM patients. The reliability, convergent and discriminant validity, and responsiveness of the MSQ were assessed.

**Results:**

CFA confirmed the currently proposed three-factor MSQ latent structure across the two studies. Good reliability was observed for all three MSQ scales, across studies and time points. MSQ scale scores strongly correlated with the scores of the Headache Impact Test-6 (HIT-6). Analysis of known-groups validity indicated that MSQ scale scores discriminated between groups of patients differing in their 28-day headache frequency were as follows <10, 10–14, and ≥15 days, and the sample-derived quartiles of the total cumulative hours of headache were as follows <140, 140 to <280, 280 to <420, and ≥420 h (*p* < 0.0001), across both studies and time points. MSQ change scores were higher in magnitude in groups experiencing greater decline in headache frequency (*p* < 0.001).

**Conclusion:**

The MSQ is a psychometrically valid tool that can be used to reliably measure the impact of migraine among CM patients.

## Introduction

Migraine attacks are characterized by severe pain and may be accompanied by nausea, photosensitivity, or other migraine-associated symptoms leading to substantial disability [1]. As a result, migraine disorder has been shown to significantly impact health-related quality of life (HRQL) both during and between attacks [2, 3]. It is expected that treatment that effectively reduces migraine-associated symptoms or the frequency of headaches will also improve patient’s HRQL. Indeed, experts have recommended [4, 5] the use of disease-specific patient-reported outcome (PRO) measures to quantify the potential benefits of treatment in migraine clinical trials.

The Migraine-Specific Quality of Life Questionnaire version 2.1 (MSQ) is a 14-item PRO instrument that measures the impact of migraine across three essential aspects of a patient’s HRQL over the past 4 weeks: role function-restrictive (RR), role function-preventive (RP), and emotional function (EF). The MSQ’s conceptual framework was developed from an expert review of the migraine literature and subsequently validated in a clinical sample of 458 migraine patients [6]. Furthermore, several studies [7–10] have further demonstrated that the MSQ possesses good psychometric properties among migraine patients.

Studies have suggested that migraine patients who experience a greater frequency of headache attacks are subjected to significantly greater levels of disability and reduced health-related quality of life [11, 12]. Differences in the frequency of migraine attacks have also been linked to clinical and pathophysiologic features of migraine. Migraine can be divided into episodic migraine (EM) and chronic migraine (CM), based on the frequency of headache days. CM is characterized by the International Classification of Headache Disorders revised criteria (ICHD-II) as experiencing 15 or more headache days per month for at least 3 months with at least 8 days per month being migraine days. CM is a subtype of chronic daily headache, and evidence suggests that CM patients experience neurological alterations even in the absence of headache, which differs from the intermittent changes noted in EM (<15 headache days/month) during headache attacks [13, 14]. Furthermore, it has been observed that CM patients exhibit lower pain thresholds when compared to patients with EM [15].

Although the MSQ has been shown to have good psychometric performance in measuring headache impact across CM and EM patients [16], the assumption that its validity is retained across these clinical subgroups has not been verified. Given the extensive use of the MSQ in migraine research and practice [6, 7, 17–19] and the emerging evidence regarding several distinguishing characteristics of CM when compared to EM patients, studies assessing the validity of the MSQ specifically in CM are needed. The current study aims to provide evidence of the psychometric properties of the MSQ using data from two clinical trials of CM patients undergoing prophylactic treatment.

## Materials and methods

### Sample

Data used in these analyses came from a total sample of 1,376 CM patients who participated in 2 studies that evaluated onabotulinumtoxinA (BOTOX^®^, Allergan, Inc., Irvine, CA) as headache prophylaxis—the Phase III REsearch Evaluating Migraine Prophylaxis Therapy (PREEMPT) trials with Botulinum Toxin Type A [20, 21]. Both PREEMPT trials were multicenter, double-blind, randomized, placebo-controlled, with a 24-week parallel-group phase followed by an open-label 32-week extension. All analyses were conducted by pooling treatment groups.

To be considered eligible for the trial, participants had to be between the ages of 18 and 65 and fulfill each of the following headache-related criteria: (1) history of migraine headache disorder meeting any of the diagnostic criteria listed in ICHD-II [22] section 1, for migraine, with the exception of “complicated migraine”; (2) ≥4 distinct headache episodes each with a duration of at least 4 h during the 4-week baseline phase; (3) ≥15 headache days during the 4-week baseline phase, with each headache day consisting of ≥4 h of continuous headache; and (4) ≥50 % of baseline headache days were migraine or probable migraine days. Headache-related exclusion criteria included any of the following criteria: (1) diagnosis of complicated migraine, basilar migraine, ophthalmoplegic migraine, or migrainous infarction; (2) use of any headache prophylactic medication within 28 days prior to screening; (3) diagnosis of chronic tension-type headache, hypnic headache, hemicrania continua, or new daily persistent headache; (4) headache attributed to another disorder (e.g., cervical dystonia, craniotomy, head/neck trauma); and (5) unremitting headache lasting continuously throughout the 4-week baseline period. In addition, participants with a Beck Depression Inventory score > 24 at week 4 baseline period were also excluded.

Following the 4-week baseline phase, patients meeting the inclusion/exclusion criteria were assigned in a blinded fashion to the study treatment in the strata of medication overuse (yes/no), as determined by the frequency of acute headache pain medication use during the baseline phase. Within each stratum, patients were randomly allocated to receive either onabotulinumtoxinA or placebo. In the double-blind phase, participants received injections with onabotulinumtoxinA at week 0 (baseline) and week 12. Only data from the 24-week double-blinded period of the two trials were used for the current study. As previously reported, the PREEMPT studies were conducted in accordance with the Declaration of Helsinki ethical principles, Good Clinical Practices, principles of informed consent, and requirements of public registration of clinical trials in the United States. The studies were approved at each site by an independent ethics committee or a local institutional review board. Written informed consent was obtained from each randomized participant prior to any study-related procedures [20, 21].

### Study measures

#### Migraine-Specific Quality of Life Questionnaire, version 2.1 (MSQ)

The 14-item MSQ is designed to measure how migraines affect and/or limit daily functioning across three domains: RR (7 items assessing how migraines limit one’s daily social and work-related activities); PR (4 items assessing how migraines prevent these activities); EF (3 items assessing the emotions associated with migraines). Participants respond to items using a 6-point scale: “none of the time,” “a little bit of the time,” “some of the time,” “a good bit of the time,” “most of the time,” and “all of the time,” which are assigned scores of 1 to 6, respectively. Raw dimension scores are computed as a sum of item responses and rescaled from a 0 to 100 scale such that higher scores indicate better quality of life.

Multiple studies have demonstrated good reliability and validity of the MSQ in subjects with migraine [6–9]. The MSQ has been administered in several efficacy trials of migraine treatment and has been has shown to be responsive to treatment effects [17, 18, 23]. Results from a study of 119 migraineurs recruited at 4 headache clinics revealed that the effect sizes of the MSQ were moderate to large at 4 and 12 weeks [24]. More recently, for each group, the minimally important differences (MIDs) were estimated for the three MSQ scales by an anchor-based approach using data from both a clinical trial and a population-based study [10].

#### The Headache Impact Test-6 (HIT-6)

The HIT-6 was adapted from the longer, Internet-based HIT [25], as a short pencil-and-paper survey assessing the impact of headache on participants’ lives in the past 4 weeks. It is a brief instrument covering a broad content of headache-related HRQL across the following domains: pain, social functioning, role functioning, vitality, cognitive functioning, and psychological distress. Each item is answered on a 5-point Likert-scale (6 = never; 8 = rarely, 10 = sometimes, 11 = very often; 13 = always). The currently recommended scoring of the HIT-6 was derived to approximate the total score obtained from the larger battery of items, using results from item response theory [26]. The final score is obtained from simple summation of the six items. The HIT-6 total score ranges between 36 and 78, with larger scores reflecting greater impact. Four groups have been derived to aid in the interpretation of HIT-6 scores: scores ≤ 49 represent little or no impact, scores between 50 and 55 represent some impact, scores between 56 and 59 represent substantial impact, and scores ≥ 60 indicate severe impact [27].

#### Migraine diary

Using a self-administered diary, participants were asked to report information on the start and stop time of any headache, headache-specific characteristics and symptoms, and use of any acute headache pain medication. A three-day “missing-recall” window was used, which allowed participants to report this information for the current date and for the 3 days immediately preceding it. If the 28-day diary had at least 20 but less than 28 days of reported data, a prorated approach was used. If a patient reported less than 20 days of headache data, the patient’s observed diary data in that particular 28-day dairy was set to missing. A headache day was defined as a day with 4 or more continuous hours of headache. A migraine day was defined as a day with 4 or more continuous hours of migraine headache (ICHD-II criteria for migraine without aura or migraine with aura). A probable migraine day is defined as a day with 4 or more continuous hours of probable migraine headache (ICHD-II for probable migraine). Information on acute headache pain medication use was used to define medication overuse.

### Statistical analyses

The psychometric evaluation of the MSQ was conducted in a sequential process. Data were first evaluated to determine the comparability of the two study samples and the adequacy of analytical approaches that may be sensitive to distributional characteristics. Next, several analyses were conducted to ensure the stability of the MSQ measurement model. Confirmatory factor analysis (CFA) was employed in order to ensure consistency with the currently proposed MSQ measurement model. Upon the verification of the stability of the MSQ measurement model, item-level psychometric indicators were examined, followed by an evaluation of the instrument’s reliability, construct validity, and ability to detect change.

#### Measurement model

The latent structure of the MSQ was examined under CFA using data collected at baseline. In addition to the currently proposed three-factor model, other alternative domain structures were fitted to test whether these would provide a better fit to the data. CFA solutions were extracted using the robust maximum likelihood (MLR) estimator in the Mplus software, version 5.1 [28]. The MLR estimator computes standard errors that are robust to non-normality. The CFA model fit was assessed using several indicators: comparative fit index (CFI), Tucker–Lewis Index (TLI), and root mean square error of approximation (RMSEA). Hu and Bentler’s guidelines [29] were used to interpret the values of CFI and TLI (≥.95), and RMSEA (<.06), indicating a good fit. Standardized factor loadings and factor correlations were also computed. High factor loadings (≥0.70) indicate convergent validity and support the hypothesized relationships between items and the corresponding latent factors (i.e., domains). Relatively lower correlations between factors indicate discriminant validity and support the domain structure of the MSQ.

#### Reliability

Indices of reliability reflect the consistency and reproducibility of scores produced by a particular measurement procedure. Internal consistency reliability was evaluated by examining the equivalence of responses within the MSQ in a single administration. Internal consistency reliability of the MSQ at baseline and week 24 was measured with three indices: (1) Cronbach’s alpha, (2) the average inter-item correlation [30], and (3) the item-total correlation after correcting for overlap (i.e., after removing the item from the total score). Cronbach’s alpha was evaluated against currently recommended criteria [31]. Item-total correlations and average inter-item correlations of 0.4 or higher were deemed indicative of good reliability [32, 33].

### Construct validity

#### Convergent validity

The convergent validity of the MSQ scores was assessed in relation to HIT-6 scores. The Pearson correlation coefficient was used to evaluate the degree of association between HIT-6 and those with MSQ scale scores. Correlation coefficients were evaluated at baseline and at week 24.

#### Known-groups validity

Construct validity was also examined using the framework of known-groups validity [34] by comparing mean MSQ scale scores across groups known to differ on a clinical criterion measure. Comparison groups were based on the following clinical indicators of CM: (1) number of headache days within a 28-day period and (2) cumulative hours of headache within a 28-day period. Drawing on classification criteria previously used in migraine research [35], participants were classified into one of three headache frequency categories based on frequency of headache days per 28 days at the primary time point Week 24: <10, 10–14, or ≥15 headache days per 28 days. In addition, four groups were formed based on quartiles of the sample’s (combined study 1 and study 2) distribution of cumulative hours of headache: (1) <140, (2) 140 to <280, (3) 280 to <420, and (4) ≥420 h. These cutoffs corresponded to an average of 5, 10, and 15 h of headache per day, respectively.

#### Responsiveness

Responsiveness is a fundamental aspect of construct validity that measures the instrument’s ability to detect changes in health status. The responsiveness of the MSQ was evaluated against changes (from baseline to week 24) in frequency of headache days. Participants were categorized according to the direction and magnitude of change in these measures. If the 28-day frequency of headache days improved (from day 0 to week 24) by at least 50 %, the subject was categorized as “much improved”; if improvement was at least 30 % but less than 50 %, the patient was considered to have “moderately improved”; if improvement was less than 30 % or if worsening was reported, the patient was classified as “minimally/not improved”. We note that very few study participants reported worsening of either frequency of headache days or cumulative hours of headache. Hence, we opted to include these patients in the same group as those reporting minimal improvement (<30 %). Our criterion of change (30 % or higher) is based on recommendations of the Task Force of the International Headache Society Clinical Trials Subcommittee [5]. F-tests obtained from the analysis of variance (ANOVA) models were used to evaluate whether the differences in mean MSQ scale change score between groups were statistically significant. The standardized response mean (SRM), evaluated as the ratio of the mean MSQ change score to its standard deviation, was evaluated to help interpret the magnitude of change across the three improvement groups defined above. A second set of analogous analyses was conducted after a term for medication overuse was included in the model.

## Results

### Sample characteristics

Table 1 presents the sample demographic and clinical characteristics, by study. Overall, study participants were primarily female, Caucasian, and had an average age of approximately 41 years. On average, participants had been experiencing frequent migraines for approximately 19 years prior to study enrollment. Based on patients’ baseline assessment, the average number of migraine days in a 28-day period was approximately 16 (19 when probable migraine days were also included). Approximately, 60 % of study participants met the criteria for medication overuse.

**Table 1:** Characteristics of study participants at baseline

Characteristics	Study 1 (*n* = 672)	Study 2 (*n* = 704)
Age (years), mean (SD)	41.6 (10.5)	41.0 (10.6)
Gender, *n* (% female)	588 (87.5)	601 (85.4)
Race, *n* (%)		
Caucasian	607 (90.3)	632 (89.8)
Black	30 (4.5)	44 (6.3)
Hispanic	29 (4.3)	17 (2.4)
Asian	3 (0.4)	4 (0.6)
Other	3 (0.4)	7 (1.0)
Migraine characteristics		
Years since frequent migraine onset, mean (SD)	20.5 (13.0)	18.0 (12.1)
Number of migraine days in a 28-day period, mean (SD)	16.5 (5.8)	16.3 (5.8)
Number of migraine/probable migraine days in a 28-day period, mean (SD)	19.1 (4.4)	18.9 (4.0)
Cumulative hours of headache in a 28-day period, mean (SD)	285.3 (114.3)	291.6 (119.6)
Analgesic medication overuse, *n* (%)	457 (68.0)	443 (62.9)
MSQ		
Total	56.6 (17.7)	54.6 (18.0)
Role function—preventive	44.6 (21.0)	43.3 (21.9)
Role function—restrictive	62.2 (16.8)	60.6 (16.9)
Emotional function	59.7 (24.1)	55.8 (24.8)
HIT-6, mean (SD)	65.6 (4.0)	65.3 (4.4)

HIT-6 varies between 36 and 78 with higher scores indicating higher headache impact. The MSQ total and scale score varies between 0 and 100 with higher scores indicating higher impact

*HIT*-*6* Headache Impact Test 6, *MSQ* Migraine-Specific Questionnaire, *SD* standard deviation

Baseline scores on the MSQ and the HIT-6 were nearly identical across the two studies. At baseline, the average HIT-6 score was approximately 65 (65.6 and 65.3 for studies 1 and 2, respectively), reflecting a severe level of headache impact [27]. Scores on the MSQ were also reflective of substantial impact on HRQL. The ranking of MSQ domains in terms of impact was consistent across the two studies, with migraine-attributable interruptions in daily activities (RP domain) reflecting the lowest impact and limitations due to migraine (RR domain) being the most severely affected of the three MSQ domain.

### Structural validity

Standard factor loadings indicated support for the three-factor model of the MSQ, with factor loadings above 0.70 for all items, except for item 12 (“*have you felt fed up or frustrated because of your migraines?*”) (Table 2). The factor loadings for item 12 ranged between 0.62 (study 1) and 0.67 (study 2). The goodness of fit indices also suggested that the three-factor model was an adequate representation of the latent structure represented by the 14 items of the MSQ (study 1/study 2: RMSEA [90 % confidence interval] = 0.06 [0.05–0.07]/0.06 [0.05–0.06]); CFI = 0.96/0.97; TLI = 0.96/0.96).

**Table 2:** Confirmatory factor analysis: completely standardized factor loadings and standard errors (SE) for the proposed three-factor measurement model for the Migraine-Specific Questionnaire

	Study 1 (*N* = 669)	Study 2 (*N* = 696)
*Role function*-*restrictive (RR)*		
1 […] have migraines interfered with how well you dealt with family, friends, and others who are close to you?	0.77 (0.02)	0.77 (0.02)
2 […] have migraines interfered with your leisure time activities, such as reading or exercising?	0.81 (0.02)	0.78 (0.02)
3 […] have you had difficulty in performing work or daily activities because of migraine symptoms?	0.86 (0.01)	0.87 (0.01)
4 […] did migraines keep you from getting as much done at work or at home?	0.82 (0.02)	0.84 (0.01)
5 […] did migraines limit your ability to concentrate on work or daily activities?	0.86 (0.01)	0.85 (0.01)
6 […] have migraines left you too tired to do work or daily activities?	0.79 (0.02)	0.79 (0.02)
7 […] have migraines limited the number of days you have felt energetic?	0.71 (0.02)	0.71 (0.02)
*Role function*-*preventive (RP)*		
8 […] have you had to cancel work or daily activities because you had a migraine?	0.84 (0.02)	0.85 (0.01)
9 […] did you need help in handling routine tasks such as every day household chores, doing necessary business, shopping, or caring for others, when you had a migraine?	0.79 (0.02)	0.79 (0.02)
10 […] did you have to stop work or daily activities to deal with migraine symptoms?	0.81 (0.02)	0.81 (0.02)
11 […] were you not able to go to social activities such as parties, dinner with friends, because you had a migraine?	0.81 (0.02)	0.82 (0.02)
*Emotional function (EF)*		
12 […] have you felt fed up or frustrated because of your migraines?	0.62 (0.03)	0.67 (0.03)
13 […] have you felt like you were a burden on others because of your migraines?	0.88 (0.02)	0.84 (0.02)
14 […] have you been afraid of letting others down because of your migraines?	0.79 (0.02)	0.86 (0.02)
Chi-square (DF)	265.80 (74)	235.23 (74)
CFI/TLI	0.96/0.96	0.97/0.96
RMSEA (90 % confidence interval)	0.06 (0.05–0.07)	0.06 (0.05–0.06)
Correlation (RR, RP)	0.91	0.87
Correlation (RR, EF)	0.74	0.68
Correlation (RP, EF)	0.71	0.72

*CFI* comparative fit index, *DF* degrees of freedom, *RMSEA* root mean square error of approximation, *TLI* Tucker–Lewis Index

Given the slightly lower factor loadings and the behavior of item-scale correlations observed for item 12 (see Table 3), two additional factor structures were fitted, using data from each study in turn. The first model differed from the three-factor MSQ model because item 12 was allowed to load on the RR factor, rather than the EF factor (it was noted that the correlations of item 12 with the EF scale were nearly identical to the correlations with the RR scale; see Table 3). Compared with the original three-factor model, this model resulted in similar goodness of fit indicators (study 1/study 2: RMSEA [90 % confidence interval] = 0.07/0.06 [0.06–0.08/0.06–0.07]), but the loading of item 12 on the RR factor was substantially lower (study 1/study 2: 0.56/0.58) than the factor loading of item 12 on the EF factor (study 1/study 2: 0.62/0.67), lending support for the inclusion of item 12 in the EF factor.

**Table 3:** Measures of internal consistency reliability for the three-factor Migraine-Specific Questionnaire measurement model, by study (baseline data)

	Item-scale correlations corrected for overlap						Cronbach’s alpha/average inter-item correlation		Cronbach’s alpha—item deleted	
	RR		RP		EF					
	Std 1	Std 2	Std 1	Std 2	Std 1	Std 2	Std 1	Std 2	Std 1	Std 2
*Role function*-*restrictive (RR)*							0.93/0.71	0.93/0.70		
1 […]**interfered** with how well you dealt with family, friends and others who are close to you?	**0.73**	**0.74**	0.64	0.63	0.59	0.56			0.92	0.92
2 […]**interfered** with your leisure time activities, such as reading or exercising?	**0.78**	**0.75**	0.70	0.65	0.54	0.48			0.91	0.92
3 […]**difficulty** in performing work or daily activities because of migraine symptoms?	**0.81**	**0.83**	0.75	0.72	0.56	0.53			0.91	0.91
4 […]**keep you** from getting as much done at work or at home?	**0.78**	**0.79**	0.69	0.71	0.58	0.54			0.91	0.91
5 […]**limit** your ability to concentrate on work or daily activities?	**0.82**	**0.82**	0.73	0.67	0.59	0.51			0.91	0.91
6 […]**left you too tired** to do work or daily activities?	**0.76**	**0.75**	0.71	0.70	0.53	0.56			0.92	0.92
7 […]**limited** the number of days you have felt energetic?	**0.69**	**0.69**	0.61	0.55	0.53	0.49			0.92	0.92
*Role function*-*preventive (RP)*							0.88/0.70	0.98/0.71		
8 […]**cancel** work or daily activities because you had a migraine?	0.72	0.72	**0.79**	**0.80**	0.50	0.54			0.84	0.85
9 […]**need help** in handling routine tasks such as every day household chores, (...) when you had a migraine?	0.71	0.65	**0.72**	**0.73**	0.55	0.59			0.86	0.87
10 […]**stop** work or daily activities to deal with migraine symptoms?	0.72	0.70	**0.74**	**0.75**	0.53	0.54			0.85	0.86
11 […]**not able to go** to social activities such as parties, (...) because you had a migraine?	0.71	0.70	**0.75**	**0.77**	0.50	0.54			0.85	0.86
*Emotional function (EF)*							0.80/0.61	0.83/0.66		
12 […]**felt** fed up or frustrated because of your migraines?	0.54	0.56	0.42	0.49	**0.54**	**0.59**			0.83	0.85
13 […]**felt** like you were a burden on others because of your migraines?	0.61	0.53	0.59	0.59	**0.73**	**0.73**			0.63	0.71
14 […]**afraid** of letting others down because of your migraines?	0.55	0.54	0.51	0.56	**0.68**	**0.75**			0.68	0.69

Bolded cells represent correlations between items and their corresponding scale total score (calculated after exclusion of the item) and are hypothesized to be highest within the corresponding row

*Std* study

It was also noted that the correlations between the three factors were high (between 0.71 and 0.91 in study 1, and between 0.72 and 0.87 in study 2; see Table 2), indicating that a single factor model might be a good representation for the MSQ latent structure. A single factor model was thus fit to the data of each study separately to examine this possibility. The single factor model resulted in poorer goodness of fit indicators study 1/study 2: RMSEA [90 % confidence interval] = 0.10 [0.09–0.11]/0.11 [0.10–0.11]); CFI = 0.91/0.88; TLI = 0.89/0.86) and lower factor loadings for the 3 items of the EF factor, when compared to the three-factor structure.

### Reliability

At baseline, Cronbach’s alpha was consistently at or above the recommended threshold for good to excellent reliability (0.80) for all 3 scales, varying between 0.80 (EF) and 0.93 (RR) for study 1, and between 0.83 (EF) and 0.93 (RR) for study 2 (Table 3). Internal consistency reliability was equally high at week 24, with Cronbach’s alpha varying between 0.90 and 0.97, across the three scales and the two studies (results not shown). The relative contribution of each item to the scale’s internal consistency was assessed by evaluating alpha-removed statistics. The magnitude of change in Cronbach’s alpha supported the notion of nearly uniform contributions of each item to its scale. The only exception occurred with the EF scale for which removal of item 12 resulted in a small increase in the value of Cronbach’s alpha (study 1/study 2 Cronbach’s alpha change = 0.03/0.02). Item-total correlations were higher than 0.40 across the two studies at both baseline and week 24, supporting the validity of each item to the total scale. Slightly lower values were observed for item 12 for which baseline item-total correlations were 0.54 and 0.59 for study 1 and 2, respectively. Correlations between item 12 and the total EF score after removing item 12 from the scale were higher at week 24 (study 1/study 2: 0.78/0.73, not shown). At baseline, the average inter-item correlation was (study 1/study 2) 0.71/0.70, 0.70/0.71, and 0.61/0.66 for the RR, RP, and EF scale, respectively. These values were slightly higher at week 24 (≥0.8, across the three scales and the two studies). Overall, using recommended interpretation guidelines, measures of reliability were homogeneously supportive of the hypothesis of consistent and reproducible MSQ scores among CM patients.

### Construct validity

#### Convergent/discriminant validity

The absolute value of correlations between MSQ scale scores and HIT-6 scores was above the recommended threshold of 0.40 for convergent validity [32] across studies and time points, ranging between 0.59 and 0.86 (Table 4).

**Table 4:** Convergent/discriminant validity: correlations between Migraine-Specific Questionnaire scale scores and scores on the Headache Impact Test-6

	Scale	Study 1		Study 2	
		Pearson	*N*	Pearson	*N*
Baseline	RR	−0.75	670	−0.78	701
	RP	−0.67	670	−0.66	702
	EF	−0.62	671	−0.59	703
Week 24	RR	−0.86	585	−0.84	646
	RP	−0.74	584	−0.74	647
	EF	−0.78	583	−0.74	647

*RR* role restrictive, *RP* role preventive, *EF* emotional function

#### Known-groups validity

Mean RR, RP, and EF scale scores differed significantly in the ANOVA model comparing patients grouped by frequency of headache days per 28 days, with and without adjusting for the presence of medication overuse (Table 5). Similarly, a decreasing trend in mean MSQ scale scores among patients with a greater number of headache hours was also observed. This trend was consistent, with the exception of the mean EF scale score among patients who experienced greater than or equal to 420 h of headache in study 2 (EF = 48.1), which was slightly higher than the mean score among patients who experienced between 280 and 420 h of headache (EF = 41.6).

**Table 5:** Known-groups validity: Migraine-Specific Questionnaire scale scores at week 24 in relation groups defined by chronic migraine clinical criterion measures

Study	MSQ scale	Cumulative hours of headache												*F* ^a^	*F* ^b^
		<140 h			140–<280 h			280–<420 h			≥420 h				
		Mean	SD	*N*	Mean	SD	*N*	Mean	SD	*N*	Mean	SD	*N*		
1	RR	61.5	21.9	282	44.8	19.8	149	41.1	19.8	91	34.8	22.2	65	46.7^c^	48.7^c^
	RP	74.8	20.6	282	61.4	21.8	149	58.1	22.9	91	52.7	25.1	64	29.7^c^	30.4^c^
	EF	65.2	28.4	280	48.4	27.7	148	42.2	27.0	91	40.5	28.5	64	27.1^c^	28.0^c^
2	RR	60.9	22.7	337	46.7	21.4	169	39.9	19.6	85	35.7	20.7	56	41.2^c^	40.8^c^
	RP	73.7	21.2	337	61.1	23.5	169	55.3	23.2	85	54.2	24.3	56	27.7^c^	27.3^c^
	EF	67.2	27.0	337	52.7	28.1	169	41.6	25.6	85	48.1	26.4	56	28.3^c^	27.8^c^

Study	MSQ scale	Number of headache days per 28 days													
		<10 days			10–14 days			≥15 days							
1	RR	63.0	23.1	235	49.4	18.7	122	39.9	20.2	230				69.8^c^	70.3^c^
	RP	75.6	21.1	235	66.5	20.5	122	56.9	23.2	229				43.0^c^	43.0^c^
	EF	66.3	29.8	233	55.0	25.4	122	42.5	27.3	228				41.5^c^	41.6^c^
2	RR	64.5	22.3	268	47.3	21.4	148	41.1	19.9	231				80.3^c^	76.0^c^
	RP	76.3	21.2	268	62.2	21.9	148	57.3	23.3	231				49.0^c^	45.7^c^
	EF	71.2	26.3	268	52.8	27.6	148	47.1	25.9	231				55.9^c^	52.5^c^

*MSQ* Migraine-Specific Questionnaire, *SD* standard deviation

^a^ *F* statistics and *p* values for between-category comparisons are from analysis of variance (ANOVA). The main effect in the ANOVA was ranked category of decrease in the clinical criterion measure (i.e., headache days or cumulative hours), where the type III sum of squares was used

^b^ *F* statistics and *p* values for between-category comparisons are from analysis of variance (ANOVA). The main effects in the ANOVA included ranked category of decrease in the clinical criterion measure (i.e., headache days or cumulative hours) and medication-overuse strata, where the type III sum of squares was used

^c^ *p* value <0.001

### Responsiveness

In both studies, all three mean MSQ scale scores were higher among patients who experienced a greater decrease from baseline in the number of headache days (Table 6). This effect was highly significant (*p* < 0.001) in both studies and for all three MSQ scales even after controlling for medication overuse. Using Cohen’s standards [36] as a guideline for interpretation, MSQ change scores indicated large and moderate effect sizes for patients who experienced ≥50 % improvement and improvement between 30 and 50 %, respectively. Change scores among the group with minimal or no improvement were small or nearly null. The RR, which was the MSQ domain most impacted at baseline, was slightly more responsive than the RP and EF scales, as indicated by greater SRM values.

**Table 6:** Responsiveness: average change in Migraine-Specific Questionnaire scale scores in relation to changes in number of headache days from baseline to week 24

Percentage improvement in headache frequency	*n*	Mean change in MSQ	SD	SRM	*F* value^a^	*p* Value^a^	*F* value^b^	*p* Value^b^
*Role restrictive*								
Study 1 (%)								
≥50	234	23.3	24.0	1.0	63.5	<0.001	63.4	<0.001
≥30 to <50	107	12.6	20.1	0.6				
<30	244	3.0	14.0	0.2				
Study 2 (%)								
≥50	269	23.1	22.4	1.0	72.9	<0.001	71.2	<0.001
≥30 to <50	116	10.6	21.8	0.5				
<30	262	2.7	15.0	0.2				
*Role preventive*								
Study 1 (%)								
≥50	234	18.5	22.2	0.8	45.4	<0.001	45.3	<0.001
≥30 to <50	107	11.2	19.0	0.6				
<30	243	1.7	16.4	0.1				
Study 2 (%)								
≥50	269	18.4	22.8	0.8	54.9	<0.001	55.4	<0.001
≥30 to <50	116	8.5	20.1	0.4				
<30	262	0.4	16.1	0.0				
*Emotional function*								
Study 1 (%)								
≥50	233	24.6	27.91	0.88	50.7	<0.001	50.6	<0.001
≥30 to <50	105	14.9	23.56	0.63				
<30	243	2.2	20.58	0.11				
Study 2 (%)								
≥50	269	26.8	26.34	1.02	74.6	<0.001	73.9	<0.001
≥30 to <50	116	11.0	24.84	0.44				
<30	261	1.8	19.97	0.09				

Percentage improvement in 28-day frequency of headache days was evaluated as

$$ \frac{{({\text{frequency}}\;{\text{at}}\;{\text{week}}\;24-{\text{frequency}}\;{\text{at}}\;{\text{baseline}})}}{{{\text{frequency}}\;{\text{at}}\;{\text{baseline}}}} \times 100 $$

*MSQ* Migraine-Specific Questionnaire, *SD* standard deviation, *SRM* standardized response mean

^a^ *F* statistics and *p* values for between-category comparisons are from analysis of variance (ANOVA). The main effect in the ANOVA was ranked category of decrease in number of headache days, where the type III sum of squares was used

^b^ *F* statistics and *p* values for between-category comparisons are from analysis of variance (ANOVA). The main effects in the ANOVA included ranked category of decrease in number of headache days and medication-overuse strata, where the type III sum of squares was used

## Discussion

The findings of the current study confirmed the appropriateness of the MSQ measurement model, the tool’s construct validity, and its ability to detect change in clinical indicators of headache, across two independent samples of CM patients undergoing prophylaxis treatment. Goodness of fit indices and strong factor loadings indicated strong model fit for the three-factor MSQ model. The convergent and discriminant validity of the MSQ were also confirmed by the finding of strong correlations (−0.59 to −0.86) with the HIT-6, a headache-specific measure. The reliability of the MSQ scales was found to be excellent, and item-level reliability statistics indicated good performance for 13 of the 14 items. Known-groups validity of all three MSQ scales was confirmed, with statistically significant differences in MSQ scale scores observed across patient groups when stratified by known clinical measures such as frequency of headache days and headache hours. Responsiveness of the MSQ scales was demonstrated by the association of significantly larger increases in MSQ scale scores with greater decreases from baseline in the frequency of headache days. All of our results were nearly identical across the two studies, providing robust evidence in favor of the MSQ’s psychometric properties among CM patients.

Based on the relatively weaker psychometric properties of item 12 and high correlations between the three latent factors, alternative factor structures were investigated but none was found to provide a better fit than the current MSQ three-factor model. Although some of our findings regarding item 12 are corroborated by results from another study [9], in our study, removal of item 12 did not result in better goodness of fit indicators. The high correlations between the three latent factors of the MSQ that were observed in the current study have been discussed in a previous study [7] where significant overlap between the RR and the RP scales was found.

Some limitations should be taken into account in the interpretation of the study’s findings.

First, the patient sample was taken from two clinical trials; therefore, generalizability to the general population of CM patients may be limited. Second, the sample is representative of those migraineurs receiving onabotulinumtoxinA as prophylaxis and may not be generalizable to other migraine treatments. Finally, consistency of the measurement model across the two studies was not tested using a formal statistical approach. Such an approach would have entailed the use of multigroup CFA to test the measurement invariance of the MSQ across the two studies. Nevertheless, the similarity of both the factor loadings and the goodness of fit indicators suggest that the results of such tests would have confirmed measurement invariance across the two studies.

No prior studies have evaluated the psychometric properties of the MSQ specifically among CM patients. The evidence presented herein, and its consistency with results from previous studies suggests that the MSQ can be used to reliably measure the impact of headache across the spectrum of headache frequency, including CM.

## Abbreviations

ANOVA: Analysis of variance
CFA: Confirmatory factor analysis
CFI: Comparative fit index
CM: Chronic migraine
EF: Emotional function
EM: Episodic migraine
HIT-6: Headache Impact Test 6
HRQL: Health-related quality of life
ICHD-II: International Classification of Headache Disorders revised criteria
MLR: Robust maximum likelihood
MSQ: Migraine-Specific Quality of Life Questionnaire
PREEMPT: Phase III REsearch Evaluating Migraine Prophylaxis Therapy
PRO: Patient-reported outcome
RMSEA: Root mean square error of approximation
RP: Role function-preventive
RR: Role function-restrictive
SRM: Standardized response mean
TLI: Tucker–Lewis Index
